
JOURNAL INFORMATION
==============================
NLM Title Abbreviation: ScientificWorldJournal
Journal ID: ScientificWorldJournal
NLM Journal ID: 101131163
Journal ID: TSWJ

The Scientific World Journal

ISSN: 2356-6140
EISSN: 1537-744X
Publisher: Wiley

ARTICLE INFORMATION
==============================
PMCID: PMC6084216
PMID: 30147552
DOI: 10.1100/tsw.2001.136
Article ID: 419436
Article version: 1
Subjects: Short Report

Longevity and Stem Cells: A Genetic Connection

Geiger Hartmut 1 *
True Jarrod M. 1
de Haan Gerald 2
Van Zant Gary 1
1 Division of Hematology/Oncology, Departments of Internal Medicine and Physiology, University of Kentucky Medical Center, 800 Rose St., Lexington, Kentucky 40536-0093, USA
2 Department of Cell Biology, University of Groningen, A. Deusinglaan 1, 9713 AV Groningen, The Netherlands
*Hartmut Geiger: ghart2@pop.uky.edu
Publication date: 2001
Electronic publication date: 2001 Jun 16
Volume: 1
First page: 77
Last page: 77
Copyright: Copyright © 2001 Hartmut Geiger et al.
Copyright year: 2001
License: This is an open access article distributed under the Creative Commons Attribution License, which permits unrestricted use, distribution, and reproduction in any medium, provided the original work is properly cited.
License URL: https://creativecommons.org/licenses/by/3.0/

Funding: National Institutes of Health AG16653 Federal Ministry of Education and Research Netherlands Organisation for Scientific Research Royal Netherlands Academy of Arts and Sciences

==============================
Miami *Nature Biotechnology* Short Reports
*TheScientificWorld* (2001) 1(S3), 77SR
ISSN 1532-2246; DOI 10.1100/TSW.2001.136

### LONGEVITY AND STEM CELLS: A GENETIC CONNECTION

Hartmut Geiger[1,*], Jarrod M. True[1], Gerald de Haan[2], Gary Van Zant[1]

[1]Division of Hematology/Oncology, Departments of Internal Medicine and Physiology, University of Kentucky Medical Center, 800 Rose St., Lexington, Kentucky 40536-0093, USA and [2]Department of Cell Biology, University of Groningen, A. Deusinglaan 1, 9713 AV Groningen, The Netherlands
* ghart2@pop.uky.edu

**INTRODUCTION**. Our understanding of the biology of organismal longevity is still limited. Using a genetic approach to identify genes and pathways that regulate longevity, we did a linked locus analysis for the trait longevity in the murine BXD recombinant inbred set of strains. Based on previous observations that pointed towards a correlation between the hematopoietic system and lifespan[1,2], we also compared these results to a linked locus analysis for multiple quantitative traits that affect the hematopoietic stem cell system in young and old animals. With this comparative method we uncover a network of pathways regulating both the hematopoietic stem cell system and longevity.

**GENETIC TOOLS**. Mapping was done with MapManager QTb28, http://mcbio.med.buffalo.edu/mmQT.html. The BXD recombinant inbred set of mouse strains (BXD RI set) is based on the two parental strains C57BL/6J (B6) and DBA/2J (DBA) and has been used for different types of quantitative trait analysis[3].

**HEMATOPOIETIC ASSAYS.** Multipotent hematopoietic stem/progenitor cells can be classified, according to their self-renewal potential, into hematopoietic stem cells (HSC), early hematopoietic progenitor cells (eHPC) and late hematopoietic progenitor cells (lHPC)[2]. The cobblestone area forming cell (CAFC) assay is an in vitro limiting dilution type cell culture assay (experimental details see[2]) and was used for the evaluation of the frequency/cycling of these different types of hematopoietic stem/progenitor cells in the bone marrow. The values for lifespan of the BXD set were taken from published data[4].

**RESULTS/DISCUSSION**. Five out of six loci linked to longevity are also linked to a trait in the hematopoietic system, showing that regulatory patterns in the hematopoietic system and lifespan rely on the same pathways (see table 1). The percentage of lHPC in cell cycle and thus turnover in the hematopoietic stem cell compartment seems to have a direct influence on lifespan. It also seems to beneficially affect longevity to have a lower number of eHPC per femur, as the comparison of linked loci indicates. Mechanisms by which numbers and turnover rates of progenitors might affect longevity are not yet known. The hematopoietic traits, measured in young animals, might be useful as predictive markers for longevity in mice. In old animals, a locus is linked to change of numbers of HSC over time, fulfilling requirements for a special “aging” locus. This locus is also linked to the trait longevity and maps to the same position as a locus that regulates cytokine-induced HSC mobilization into the bloodstream. We therefore speculate that mobilization of HSC might also serve as a stem cell source for other tissues and by this mechanism prolongs lifespan. The influence of a stem cell system on longevity therefore may not be restricted to the hematopoietic stem cell system. Congenic animals, generated by the speed congenic approach, have confirmed the phenotypes of several of the mapped loci. Stem cells influence, through their capacity of replacing differentiated cells that are lost, organ homeostasis and thus function. We conclude, based on our comparative mapping data, that these “supportive qualities” of stem cell systems influence longevity.

Table 1 Comparative linked locus list for multiple quantitative trait mapping in the BXD recombinant inbred set

| linked locus | | trait | | | | | | | |
|---|---|---|---|---|---|---|---|---|---|
| | | Lifespan | Cycling of lHPCs | eHPCs/ femur | HSCs/ femur | eHPCs/femur old animals | HSCs/femur old animals | Change eHPCs/femur with age | Change HSCs/femur with age |
| Chr 1 | (38 cM) | | | B++ | B+++ | | | | |
| | (100 cM) | | | | D+ | | | | |
| Chr 2 | (70 cM) | D+ | | | | B++ | B+++ | B+ | B++ |
| Chr 3 | (24 cM) | | | | D++ | | | | |
| Chr 4 | (42 cM) | D+ | | | | | | | |
| Chr 5 | (20 cM) | | | | D++ | | | | |
| Chr 7 | (0.5 cM) | B+++ | D++ | | | | | | |
| | (8 cM) | B+ | | D+ | | | | | |
| | (25 cM) | B+ | | D+ | | | | | |
| | (50 cM) | | | D++ | | D+++ | | | |
| Chr 11 | (28 cM) | B+ | D+++ | | | | | | |
| Chr 14 | (13 cM) | | | | | D++ | | | |
| Chr 15 | (59 cM) | | | | | B+ | | B+ | |
| Chr 16 | (27 cM) | | | | D+ | D++ | D++ | D++ | |
| Chr.X | (51 cM) | | | | B++ | | | D+ | D++ |

Mapped were the traits lifespan, percentage of late hematopoietic progenitor cells (lHPCs) in cycle, number per femur of early hematopoietic progenitor cells (eHPCs) and hematopoietic stem cells (HSCs) in young (<3 months) and old (>18 months) animals and the percentage of change of numbers per femur in young versus old animals. In the first column all linked loci that were mapped with an at least suggestive linkage value are shown with their centimorgan (cM) position. The type of capital letter indicates which segment (either DBA/2 (D) or C57BL/6 (B) derived) is responsible for a higher trait value (lifespan: longer lifespan, change with age: positive change). The black color indicates no overlap linkage with other traits, the blue color indicates overlap only within hematopoietic traits and the red color indicates overlap linkage between lifespan and the hematopoietic traits. Used were either first order mapping results or results based on controlling for other loci. The linkage analysis for lifespan was based on trait values of 22 strains, the linkage analysis for the hematopoietic traits was based on trait values of all then available 26 strains, and the linkage analysis for the hematopoietic traits in old animals included trait values of 20 strains. The BXD marker database contains 319 marker loci with a complete strain distribution pattern, providing on average a 3.75 cM mapping resolution. Based on a 5000 permutation test, for all linked loci the suggestive, significant and highly significant LRS values were individually calculated: +: suggestive, ++: higher than suggestive, and up to significant and +++: above significant.

**ACKNOWLEDGEMENTS.** This work was supported by NIH grant AG16653 (to GVZ), a fellowship of the Deutsche Akademie der Naturforscher Leopoldina funded by the Bundesministerium für Bildung und Forschung (to HG) and a fellowship of the Netherlands

Organization for Scientific Research (NWO) and of the Royal Netherlands Academy of Arts and Sciences (KNAW) (to GdH).

**REFERENCES.**

1. Van Zant, G., Holland, B.P., Eldridge, P.W., and Chen, J.J. (1990) J. Exp. Med. 171, 1547-1565
2. de Haan, G. and Van Zant, G. (1997) J. Exp. Med. 186, 529-536
3. Taylor, B.A. et al. (1999) Mamm. Genome 10, 335-348
4. Gelman, R., Watson, A., Bronson, R., and Yunis, E. (1988) Genetics 118, 693-704
